# Йодный статус населения в странах Европейского региона ВОЗ (сокращенный перевод отдельных разделов доклада Европейского бюро ВОЗ)

**DOI:** 10.14341/probl13611

**Published:** 2025-09-14

**Authors:** Г. А. Герасимов

**Affiliations:** Глобальная сеть по йоду; Iodine Global Network

**Keywords:** йод, потребление йода, дефицит йода, концентрация йода в моче, йодирование соли, концентрация йода в молоке, заболевания щитовидной железы, Iodine, iodine intake, iodine deficiency, urinary iodine concentration, salt iodization, iodine concentration in milk, thyroid disease

## Abstract

This review is an abridged translation of selected chapters of the report “Prevention and control of iodine deficiency in the WHO European Region: adapting to changes in diet and lifestyle”, published by the WHO Regional Office for Europe and the Iodine Global Network (IGN) in 2024. Iodine deficiency, especially mild iodine deficiency, remains a widespread problem in the WHO European Region. Since the last WHO report on iodine deficiency in the Region 15 years ago, much new data on iodine status has become available, especially for vulnerable populations. This review presents data on the iodine status of the population in 53 WHO European Member States (and Kosovo), the adverse effects of mild iodine deficiency and the effectiveness of salt iodization in preventing iodine deficiency. Mainly due to progress in salt iodization, the number of countries with iodine deficiency has decreased from 23 in 2003 to 2 in 2023. Mandatory salt iodization ensures adequate iodine intake in all population groups, with the exception of a few countries where these programs are poorly implemented. The positive cost-benefit ratio for preventing mild iodine deficiency in the European Region is plausible given the high prevalence of thyroid disease and the low cost of salt fortification programs.

## 1. ВВЕДЕНИЕ

В 2024 г. Европейское региональное бюро ВОЗ и Глобальная сеть по йоду (ГСЙ) опубликовали доклад под названием «Профилактика и контроль дефицита йода в Европейском регионе ВОЗ: адаптация к изменениям в питании и образе жизни». Поскольку оригинальный доклад был опубликован только на английском языке [1], настоящий обзор литературы представляет собой сокращенный перевод на русский язык отдельных разделов упомянутого доклада1, содержащих информацию о методах оценки и мониторинга йодного статуса населения (Глава 5), йодном статусе отдельных групп населения стран Европейского региона ВОЗ (Глава 7) и положительном экономическом эффекте профилактики дефицита (Глава 8). Обзоры с переводом остальных глав доклада на русский язык планируется опубликовать в других научных медицинских журналах.

## 2. СБОР ДАННЫХ ДЛЯ ДОКЛАДА

В этом обзоре представлены данные о йодном статусе населения в 53 государствах — членах Европейского региона ВОЗ и Косово2 (всего — 54 страны). Национально репрезентативные данные по странам, основанные на оценке медианной концентрации йода в моче (КЙМ) у школьников, взрослых лиц и беременных женщин были получены из различных источников, описанных ниже. Поиск информации был проведен в базе данных ВОЗ по микронутриентам [2]. В период с января 2022 г. по январь 2024 г. был проведен всесторонний систематический поиск литературы в MEDLINE, EMBASE, Web of Science и Scopus для выявления соответствующих новых исследований с использованием поисковых терминов «дефицит йода», «концентрация йода в моче», «мониторинг». Был проведен ручной поиск информации в списках ссылок оригинальных статей и обзоров литературы. Запросы на данные по отдельным странам были отправлены в Региональное бюро ВОЗ и национальным координаторам ГСЙ в странах региона.

Для каждой страны были отобраны самые последние данные по медианной КЙМ (мКЙМ) для трех групп населения: детей школьного возраста и подростков (6–15 лет), взрослых (возраст более 15 лет, женщин и мужчин) и беременных женщин. Выбранные исследования должны были быть проведены в период с 2008 по 2023 гг. и быть репрезентативными на национальном уровне. При отсутствии национальных данных использовались региональные данные первого административного уровня (области, провинции и т.п.) в том случае, если выборка населения из региона являлась однородной с остальной частью населения.

Минимальный размер выборки для анализа данных мКЙМ (разовые или суточные образцы мочи) составлял не менее 100 человек. Для анализа йода в моче должны были быть использованы методы Санделла-Кольтхоффа и масс-спектрометрия с индуктивно связанной плазмой (МС), а результаты представлены в виде медианной, арифметической и/или геометрической средней КЙМ, выраженные в мкг/л, а при доступности также межквартильный размах (IQR) и/или доверительный интервал (CI).

## 3. ИНДИКАТОРЫ ДЛЯ ОЦЕНКИ ЙОДНОГО СТАТУСА

## 3.1. Концентрация йода в моче

Йодный статус для каждой группы населения определялся на основе мКЙМ и классифицировался либо как дефицит йода (легкий, умеренный и тяжелый), либо как оптимальный, либо как избыточный с риском неблагоприятных последствий для здоровья [3] (табл. 1).

**Table:** Таблица 1. Эпидемиологические критерии оценки йодного статуса населения на основе медианной концентрации йода в моче [3] a Термин «избыточный» означает превышение количества, необходимого для профилактики дефицита йода. Хроническое воздействие избытка йода связано с повышенной распространенностью аутоиммунных заболеваний щитовидной железы, гипотиреоза, гипертиреоза и зоба.

Уровень потребления	Йодный статус	Школьники	Взрослые	Беременные женщины
Медианная КЙМ (мкг/л)
Недостаточный (дефицит)	Тяжелый дефицит	<20 мкг/л	Не определена	Не определена
	Умеренный дефицит	20–49 мкг/л	Не определена	Не определена
	Легкий дефицит	50–99 мкг/л	<100 мкг/л	<150 мкг/л
Адекватный	Оптимальный	100–299 мкг/л	≥100 мкг/л	150–249 мкг/л
Избыточныйa	Риск неблагоприятных последствий для здоровья	≥300 мкг/л	Не определена	≥500 мкг/л

Поскольку более 90% йода, поступающего в организм с пищей, выводится с мочой в течение 24 часов после потребления, мКЙМ отражает текущее потребление йода из всех пищевых источников. Дефицит и избыток йода среди населения определяется путем сравнения мКЙМ у населения с пороговыми значениями, определенными ВОЗ [3] и представленным в таблице 1. Однако до настоящего времени пороговые значения мКЙМ для взрослых, беременных женщин и младенцев остаются предметом дискуссии [4], а эксперты ВОЗ занимаются их пересмотром. Предполагается, что для надежной оценки йодного статуса на основе мКЙМ в конкретной популяции необходимо исследование приблизительно 500 человек [5]. Из-за высокой вариабельности потребления йода показатели КЙМ не могут использоваться для оценки индивидуального потребления йода или для диагностики дефицита йода [6]. В период лактации концентрация йода в грудном молоке может быть более надежным индикатором йодного статуса у младенцев, чем мКЙМ [4].

Йодный статус можно исследовать в любой группе населения, но ВОЗ рекомендует наблюдение за детьми школьного возраста, взрослыми и/или беременными женщинами [3]. Мониторинг в одной и той же группе населения с течением времени может помочь лучше выявлять изменения в потреблении йода и облегчить анализ тенденций. мКЙМ традиционно исследовалась у детей в возрасте 6–12 лет, поскольку наблюдения на базе школ были логистически наиболее осуществимы, а йодный статус у детей считался репрезентативным для общей популяции [3][7]. Однако в последние годы условия в Европейском регионе изменились. Мотивация школ, родителей и детей к участию в популяционных исследованиях стала сложной задачей, что приводит к большому числу отказов и искажает репрезентативность исследования и интерпретацию результатов. Кроме того, по ряду причин, указанных ниже, и исследования среди детей могут не быть репрезентативными для общей популяции.

Сведения о дефиците йода во многих странах Региона побудили проводить исследования мКЙМ у беременных женщин, т.е. группы населения, особенно уязвимой к дефициту йода. Логистика исследований йодного статуса йода у беременных женщин может был облегчена тем, что они регулярно сдают мочу на анализ в ходе дородового наблюдения в медицинских учреждениях.

Влияние объема мочи

Содержание йода в разовых образцах мочи может различаться в зависимости от состояния гидратации человека на момент сбора образца. Низкие объемы мочи могут переоценивать потребление йода и маскировать дефицит йода, тогда как большие объемы могут недооценивать потребление. Нынешний порог мКЙМ, определяющий адекватное потребление йода (≥100 мкг/л), изначально был определен для детей на основе нормы физиологический потребности (НФП) в йоде в 120 мкг/день, 90% экскреции йода с мочой и ее среднего объема мочи в 1,0 л в день. Медианный порог КЙМ для взрослых в 100 мкг/л был рассчитан на основе НФП в 150 мкг/день и среднего объема мочи 1,5 л/день [3]. Однако несколько недавних европейских исследований сообщают о более высоком объеме мочи у взрослых, составляющем около 1,9–2,1 л/день [8][9]. При объеме мочи около 2 л/день мКЙМ, соответствующая НФП для йода в 150 мкг/день, будет составлять 70 мкг/л, что ниже текущего порога адекватного потребления йода, рекомендуемого ВОЗ (табл. 1). При таком же объеме мочи у беременных женщин мКЙМ всего лишь 110 мкг/л может соответствовать адекватному потреблению йода ≥250 мкг/день. Следовательно, у взрослых лиц и беременных женщин мКЙМ ниже пороговых значений (соответственно, 100 мкг/л и 150 мкг/л) не обязательно указывает на дефицит йода, что имеет значение для интерпретации данных.

## 3.2. Концентрация тиреоглобулина (ТГ) в крови

Уровень ТГ в крови является индикатором йодного статуса в популяции, чувствительным к повышению активности щитовидной железы (ЩЖ) и/или увеличению ее объема. ТГ синтезируется фолликулярными клетками в ЩЖ и является предшественником для выработки тиреоидных гормонов. Уровни ТГ в крови при адекватном потреблении йода являются низкими, но увеличиваются при дефиците и избытке йода и нормализуются в ответ на восполнение дефицита йода. Однако повышенная концентрация ТГ не является специфичной для дефицита йода, и роль других факторов пока не полностью изучена. Концентрация ТГ может быть измерена в сыворотке, плазме или «сухих пятнах» цельной крови. Референтные диапазоны для ТГ различаются между аналитическими методами. Кроме того, антитела к ТГ могут мешать точному анализу. При адекватном потреблении йода в популяции частота повышенного уровня ТГ должна быть менее 3% [3][10].

## 3.3. Неонатальный ТТГ

В большинстве стран Европейского региона все новорожденные проходят плановый скрининг на врожденный гипотиреоз путем измерения ТТГ в образцах цельной крови из сухого пятна на фильтровальной бумаге, собранных через 2–5 дней после рождения. Целью неонатального скрининга является выявление новорожденных с врожденным гипотиреозом и начало незамедлительного лечения препаратами тироксина для предотвращения необратимой задержки развития нервной системы. Врожденный гипотиреоз подозревается при повышении уровня ТТГ более 20 мМЕ/л, но некоторые страны используют более низкие пороговые значения в зависимости от используемого метода. Дефицит йода во время беременности и/или после рождения может увеличивать оборот йода в ЩЖ младенца, что приводит к ее гиперстимуляции и повышению уровня неонатального ТТГ. Частота встречаемости повышенного неонатального ТТГ (>5 млМЕ/л) у более, чем 3% новорожденных может указывать на дефицит йода [3][11]. Хотя неонатальный ТТГ может быть хорошим показателем наличия умеренного или тяжелого дефицита йода во время беременности, чувствительность этого индикатора при легком дефиците йода считается низкой [11]. Тенденции в уровне неонатального ТТГ у новорожденных с течением времени могут отражать изменения в йодном статусе и потреблении йода среди населения Европейского региона.

## 3.4. Надзор за заболеваниями щитовидной железы

Некоторые страны Европейского региона ВОЗ (например, Армения, Азербайджан, Беларусь, Кыргызстан, Российская Федерация, Швеция и Украина) собирают данные о заболеваемости патологиями ЩЖ, связанными с дефицитом йода (табл. 2). Рутинный сбор сведений по заболеваемости зобом встроен в существующие системы мониторинга показателей здоровья и не требует дополнительных финансовых ресурсов или организационных усилий. В Беларуси после успешного внедрения принятых в 2001 г. требований по обязательному использованию йодированной соли в пищевой промышленности мКЙМ увеличилась с 68 мкг/л до 191 мкг/л [12], а заболеваемость зобом в период с 1998 по 2007 гг. снизилась почти в четыре раза (рис. 1). В Российской Федерации при низком охвате населения йодированной солью заболеваемость зобом у детей (0–14 лет) оставалась стабильной в период с 2009 по 2015 гг. [13]. В Швеции было зарегистрировано адекватное потребление йода у школьников (мКЙМ 125 мкг/л [14]), и в период с 1998 по 2018 гг. не было зарегистрировано ни одного случая «йододефицитного зоба (E01)» у детей и подростков. Однако тенденции заболеваемости зобом следует интерпретировать с осторожностью, поскольку они в большей степени могут зависеть от существующей клинической практики выявления зоба и не отражать изменения в йодном статусе населения.

**Table:** Таблица 2. Болезни щитовидной железы, связанные с йодной недостаточностью, и сходные состояния (E01). МКБ-10* * International Statistical Classification of Diseases and Related Health Problems. 2019. https://icd.who.int/browse10/2019/en.

Код по МКБ-10	Название заболевания
E.01.0	Диффузный (эндемический) зоб, связанный с йодной недостаточностью
E.01.1	(Много)узловой (эндемический) зоб, связанный с йодной недостаточностью
E.01.2	Зоб (эндемический), связанный с йодной недостаточностью, неуточненный
E.01.8	Другие болезни щитовидной железы, связанные с йодной недостаточностью

**Figure:**
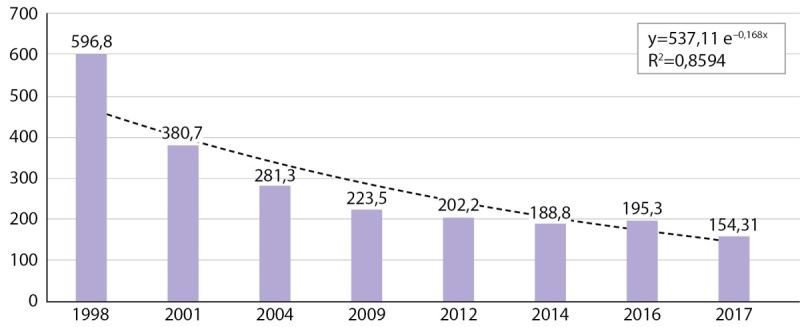
Рисунок 1. Динамика заболеваемости диффузным зобом (E01.0 и Е04.0) у детей и подростков в Республике Беларусь за период с 1998 по 2017 гг. [12].

## 4. ЙОДНЫЙ СТАТУС В ЕВРОПЕЙСКОМ РЕГИОНЕ

Эффективность национальных стратегий, направленных на профилактику дефицита йода, следует оценивать в репрезентативных популяционных исследованиях мКЙМ в идеале каждые пять лет [3]. В течение 2008–2023 гг. национальные репрезентативные исследования, проведенные среди детей в возрасте 6–15 лет, взрослых и/или беременных женщин, были доступны в 38 из 54 стран Региона.

Национально репрезентативные исследования мКЙМ среди детей школьного возраста были доступны в половине государств, но многие (29%) из них старше 10 лет. Несколько стран (например, Таджикистан, Узбекистан) оценивали мКЙМ среди детей дошкольного возраста (6–59 месяцев) в исследованиях на уровне домохозяйств вместе с женщинами репродуктивного возраста, но эти данные не были включены в данный обзор. У взрослых лиц йодный статус (мКЙМ в разовой или суточной моче) был оценен в 22 из 54 стран. В Кыргызстане, Латвии, Литве, Таджикистане и Украине национальные репрезентативные исследования среди взрослых были выполнены в течение последних пяти лет. Однако в 10 из 22 стран данные мКЙМ старше 10 лет. С 2008 по 2023 гг. в 24 странах Региона были проведены репрезентативные исследования йодного статуса у беременных женщин, большинство (18) были проведены в течение последних 10 лет. В 17 странах Региона отсутствуют данные национальных репрезентативных мКЙМ для какой-либо группы населения [1].

Потребление йода детьми школьного возраста в целом адекватно в 26 из 28 стран региона, за исключением Германии и Израиля. Данные мКЙМ у взрослых лиц (>15 лет) указывают на адекватное потребление йода в 12 из 22 государств, но в 8 странах (Швейцария, Италия, Бельгия, Германия, Литва, Финляндия, Украина и Швеция) мКЙМ была ниже 100 мкг/л. Однако в 3 странах (Швейцария, Литва и Италия) суточный объем мочи составлял 1,9–2,1 л/день [8][9], что может привести к недооценке йодного статуса с использованием порогового значения мКЙМ в 100 мкг/л. Из 24 стран, для которых имелись репрезентативные данные мКЙМ у беременных женщин, потребление йода было достаточным в 9 и недостаточным в 15 странах.

Таким образом, репрезентативные исследования мКЙМ в странах Европейского региона ВОЗ, представленные в данном обзоре, показали значительное улучшение йодного статуса среди детей школьного возраста: количество стран с дефицитом йода сократилось с 23 в 2003 г. до 2 в 2023 г.

В 2023 г. ни одна страна в Европейском регионе ВОЗ не была затронута умеренным или тяжелым дефицитом йода или имела его избыточное потребление. Дети школьного возраста имеют легкий дефицит йода в двух странах: Израиле, где ранее не было данных о мКЙМ, и в Германии, где йодный статус за последние годы ухудшился. Региональные обследования в Российской Федерации и национальное обследование на Украине в целом не показали улучшения йодного статуса населения. Недавнее национальное исследование в Таджикистане обнаружило низкую мКЙМ у детей в возрасте 6–59 месяцев, что также предполагает ухудшение потребления йода [1].

## 4.1. Влияние йодирования соли на йодный статус у различных групп населения

## 4.1.1. Обязательное йодирование соли

В период с 2008 по 2023 гг. йодный статус был оценен в 16 из 21 страны Европейского региона с обязательным йодированием соли как в домохозяйствах, так и для производства пищевых продуктов промышленного производства (ППП). Уровень йода в соли в этих странах варьировался от 15 до 65 мг/кг. Во всех 13 странах этой группы мКЙМ у школьников указывала на адекватное потребление йода (табл. 3). У взрослых йодный статус был адекватным в 5 странах, слегка повышенным — в одной стране (Армения). В Литве мКЙМ у взрослых была ниже порогового значения в 100 мкг/л, но потребление йода оценивалось примерно в 200 мкг/день с учетом высокого среднего объема мочи (2,1 л/день) [8].

**Table:** Таблица 3. Медианная КЙМ (мкг/л) у школьников (6–15 лет), взрослых (более 15 лет) и беременных женщин в странах Европейского региона ВОЗ при обязательном, добровольном йодировании соли или отсутствие требований по ее йодированию [1] Сокращения: CI — доверительный интервал; IQR — межквартильный размах.

Страны региона	Группы населенияМедианная концентрация йода в моче (мкг/л)
Школьники(6–15 лет)	Взрослые(более 15 лет)	Беременные женщины
Страны с обязательным йодированием соли (n=21)
1. Грузия	298 (IQR: 224, 374)	–	211
2. Румыния	255	–	131
3. Хорватия	251 (IQR: 157, 371)	–	–
4. Армения	242 (IQR: 203, 289)	311 (IQR: 244, 371)	226 (IQR: 209, 247)
5. Северная Македония	236	–	168
6. Болгария	182 (IQR: 119, 245)	165	170 (95% CI: 161, 177)
7. Кыргызстан	175 (95% CI: 172, 190)	167 (95% CI: 158, 175)	180 (95% CI: 146, 198)
8. Черногория	173	–	133.3
9. Косово	149 (95% CI: 144, 153)	–	183
10. Албания	136 (95% CI: 142, 161)	–	–
11. Азербайджан	135	–	151
12. Узбекистан	> 100	141 (95% CI 132, 151)	117 (95% CI: 102–140)
13. Казахстан	–	204	170
14. Таджикистан	–	122 (IQR 89, 149)	–
15. Литва	–	96	–
16. Турция	–	–	94 (IQR: 52, 153)
17. Босния и Герцеговина	–	–	–
18. Сербия	–	–	–
19. Словакия	–	–	–
20. Словения	–	–	–
21. Туркменистан	–	–	–
Страны с обязательным йодированием соли для определенных продуктов питания и/или каналов сбыта продуктов (n=9)
1. Молдова	204 (IQR: 130, 302)	136	173
2. Беларусь	191	–	121
3. Италия	124 (IQR: 77, 188)	46 (IQR: 23-88)	–
4. Австрия	127	154	–
5. Польша	120 (95% CI 74, 166)	–	112 (95% CI 98, 124; IQR 44, 179)
6. Португалия	106	–	–
7. Дания	–	–	–
8. Венгрия	–	–	–
9. Российская Федерация	–	–	–
Страны с добровольным йодированием соли (n=13)
1. Чехия	248	129	98
2. Испания	173 (95% CI:118–237)	117 (IQR: 69, 165)	–
3. Швейцария	127 (95% CI: 119, 140; IQR 87, 194)		97 (95% CI: 90, 106; IQR: 45, 187)
4. Бельгия	113 (95% CI: 110, 117; IQR: 72, 154)	94 (IQR: 68, 133)	124 (IQR: 73, 213; 95% CI 118, 131)
5. Латвия	107 (IQR: 61, 154)	60 (IQR 38-94)	69 (IQR 54, 93)
6. Германия	89 (IQR: 55, 120)	54 (IQR: 25, 83)	–
7. Греция	–	114	127
8. Финляндия	–	96 (IQR: 55, 138)	–
9. Украина	–	90 (95% CI: 84, 96)	–
10. Швеция	–	74	101 (95% CI: 95, 108; IQR: 61, 182)
11. Норвения	–	–	79 (IQR: 47-132)
12. Франция	–	–	–
13. Нидерланды	–	–	–
Страны с отсутствием регулирования по йодированию соли (n=11)
1. Исландия	200 (IQR: 90, 320)	–	89 (95% CI: 42, 141)
2. Великобритания	149	106	–
3. Ирландия	111 (IQR: 72, 165)	107 (IQR: 70, 161)	–
4. Израиль	83 (IQR: 52, 127)	–	61 (IQR: 36, 97)
5. Андорра	–	–	–
6. Кипр	–	–	–
7. Эстония	–	–	–
8. Люксембург	–	–	–
9. Мальта	–	–	–
10. Монако	–	–	–
11. Сан-Марино	–	–	–

У беременных женщин мКЙМ ниже порогового значения 150 мкг/л в Черногории (уровень обогащения соли йодом был впоследствии повышен), Румынии, Турции и Узбекистане. В Узбекистане и Таджикистане существуют проблемы с производством качественной йодированной соли и низким ее охватом домохозяйств и пищевой промышленности. Данные по йодному статусу населения, отвечающие требования этого обзора, отсутствуют для Боснии и Герцеговины, Сербии, Словакии, Словении и Туркменистана [1].

## 4.1.2. Обязательное йодирование соли для определенных продуктов питания и/или каналов сбыта

Девять стран Европейского региона ВОЗ имеют обязательную нормативную базу по йодированию соли для определенных продуктов питания и/или общественного питания (учреждения образования, здравоохранения и социального обеспечения). В большинстве из них потребление йода в целом является адекватным у школьников, хотя оно, как правило, ниже, чем в странах с обязательным йодированием соли (табл. 3). В странах с обязательным использованием йодированной соли в ППП (Беларусь) или в хлебопекарной промышленности (Австрия и Молдова) имеется адекватное потребления йода. В Италии наличие йодированной соли в продуктовых магазинах и супермаркетах является обязательным, тогда как нейодированная соль может продаваться по запросу покупателя. Закон разрешает, но не требует использование йодированной соли в пищевой промышленности и общественном питании, включая школьные столовые. Недавнее общенациональное исследование, однако, показало, что 78% школьных столовых в Италии использовали йодированную соль, и потребление йода у детей было достаточным [9].

В Российской Федерации и Португалии йодированная соль должна использоваться в общественном питании (учреждения образования, здравоохранения и социального обеспечения), но охват домохозяйств йодированной солью в них, как правило, невысок (менее 20%). В Российской Федерации никогда не проводились общенациональные исследования йодного статуса, однако данные региональных исследований, проведенных в 2008–2023 годах, показывают, что мКЙМ у школьников и беременных женщин существенно различается между регионами страны. Йодный статус был оптимальным в крупных городах (Москва, Новосибирск, Санкт-Петербург, Тюмень), тогда как в других регионах, особенно в сельской местности, он был в основном недостаточным [15]. Новые санитарные нормы и правила, принятые в 2020 г., требуют обязательного использования йодированной соли в школьных столовых и детских садах, а также в других социальных и образовательных учреждениях. В Польше йодирование соли является обязательным только для розничной торговли, а для производства ППП является добровольным и ограниченным. В Молдове уровень йодирования соли был повышен (до 25–40 мг/кг) в 2022 г., а йодирование соли стало обязательным для хлебопекарной промышленности и общественного питания. Дания пересмотрела уровень йода в соли с 13 до 20 мг/кг в 2019 г., но новые данные по мКЙМ пока отсутствуют [1].

## 4.1.3. Добровольное йодирование соли

Йодирование соли является добровольным в 13 странах Европейского региона ВОЗ. В 6 странах подтвержден адекватный йодный статус как минимум в одной группе населения (табл. 3). Однако в остальных странах мКЙМ колеблется вокруг порогового значения у взрослых (100 мкг/л) и/или у беременных женщин (150 мкг/л), что свидетельствует о пограничной недостаточности йода. Во всех 7 странах с доступными данными мКЙМ у беременных женщин составляла менее 150 мкг/л (табл. 3). По сравнению со странами с обязательным йодированием соли, в этой группе стран был более низкий охват домохозяйств йодированной солью и ее использование в производстве ППП.

Несколько стран этой группы сообщили о снижении потребления йода за последние 10–15 лет. Так, в Германии потребление йода снизилось у детей и в настоящее время является недостаточным, что связано с низкими продажами йодированной соли и недостаточным ее использованием в ППП [16]. Возможно, такая же тенденция существует в других странах в добровольным йодированием соли. На Украине недавнее национальное исследование выявило неадекватный йодный статус у взрослых (мКЙМ 90 мкг/л) [17]. На момент составления настоящего обзора производство соли на Украине было приостановлено, а пищевая соль импортируется в основном из Польши, Румынии и Турции).

## 4.1.4. Отсутствие регулирования по йодированию соли

В четырех странах (Исландия, Ирландия, Израиль и Великобритания) не существует нормативных документов по йодированию соли. Население Израиля подвержено легкому дефициту йода, тогда как в трех других странах потребление йода в целом достаточно. Данные свидетельствуют о том, что в странах Западной Европы, помимо йодированной соли, йод потребляется с молоком и молочными продуктами [1]. Население Исландии исторически получало достаточное количество йода благодаря высокому потреблению морской рыбы. Однако в связи с изменениями в структуре питания потребление йода остается достаточным у детей, но снижено у беременных женщин. В Ирландии мКЙМ также указывает на недостаточное потребление йода. В Израиле молоко и молочные продукты содержат йод, но их потребление является недостаточным для обеспечения адекватного потребления йода [18]. В Великобритании у детей (4–18 лет) и взрослых (19–64 года) йодный статус является адекватным, однако ряд региональных исследований указывают на дефицит йода у беременных (мКЙМ<150 мкг/л).

## 4.1.5. Равенство в потреблении йода

Во всем мире дефицит йода более распространен в географически отдаленных районах и среди групп с низким доходом, особенно в странах с ограниченным доступом к йодированной соли. Такая картина наблюдается в некоторых странах Восточной Европы и Центральной Азии. Более низкая мКЙМ была зарегистрирована в сельской местности по сравнению с городскими жителями у женщин репродуктивного возраста в Узбекистане и у беременных женщин в Молдове. В Узбекистане мКЙМ у женщин была адекватной в городских районах (148 мкг/л), тогда как у женщин, проживающих в Наманганской и Самаркандской областях, а также в беднейших домохозяйствах по всей стране, мКЙМ была ниже оптимальной [19]. Напротив, в Северной Македонии, где высокие уровни охвата домохозяйств адекватно йодированной солью поддерживаются на протяжении десятилетий, не было обнаружено статистически значимых различий в мКЙМ между детьми из домохозяйств с низким и высоким доходом [1]. В Италии наблюдалась противоположная тенденция: мКЙМ среди детей школьного возраста была ниже в городских районах по сравнению с сельскими [9].

## 5. ПОЛОЖИТЕЛЬНОЕ ЭКОНОМИЧЕСКОЕ ВОЗДЕЙСТВИЕ ПРОФИЛАКТИКИ ДЕФИЦИТА ЙОДА

Низкое содержание йода в почве в странах Европы было причиной тяжелого дефицита йода в XIX веке. Сегодня, несмотря на неоптимальное потребление йода во многих странах Региона, потребление йода улучшилось по сравнению с 1980-ми годами ХХ века, когда зоб был эндемичным, а субклинический гипотиреоз у новорожденных был частым явлением во многих странах. Поскольку природный дефицит йода будет сохраняться, программы обогащения соли йодом требуют постоянной приверженности со стороны правительств стране Региона.

Коррекция дефицита йода экономически эффективна, если затраты на программу обогащения ниже, чем расходы, связанные с последствиями дефицита. Негативное экономическое воздействие наиболее очевидно в группах населения с тяжелым дефицитом йода и высокой распространенностью эндемического зоба, нейро-когнитивных нарушений и тяжелой умственной отсталости, и предполагаемое соотношение затрат и выгод от обогащения йодом в этом случае составляет от 1:26 до 1:400. В 2008 г. «Копенгагенский консенсус» в составе ведущих мировых экспертов в области экономики, руководствуясь соотношением экономических затрат и выгод, поставил йодирование соли на 3-е место в списке из более чем 30 инициатив по содействию глобальному развитию, исходя из ее стоимости всего 0,05 долл. США на человека в год [20].

В отличие от тяжелого дефицита йода, последствия его легкого дефицита для здоровья человека определены менее четко, что влияет на точность оценок экономической эффективности йодной профилактики. Основным аргументом в пользу оптимизации потребления йода у населения с легким дефицитом йода является снижение расходов здравоохранения за счет профилактики развития узловых заболеваний щитовидной железы, зоба и гипертиреоза. В Швейцарии заболеваемость гипертиреозом значительно снизилась после коррекции легкого дефицита йода, а в Дании распространенность как одиночных, так и множественных узлов ЩЖ была ниже через 11 лет после начала обязательного обогащения соли йодом. Немногие исследования, оценивающие экономическое влияние легкого дефицита йода в Европейском регионе, указывают на общественную выгоду от йодной профилактики [21].

Дефицит йода является одним из многих факторов риска заболеваний ЩЖ и — потенциально — умственной отсталости. Создание Европейского регионального реестра заболеваний ЩЖ, связанных с дефицитом йода, необходимо для более точной оценки затрат и выгод коррекции легкого дефицита йода. Отсутствие данных о распространенности дефицита йода является еще одной проблемой для надежного анализа затрат и выгод. Недавняя оценка с использованием модели, разработанной для оценки преимуществ и недостатков профилактики дефицита йода в Германии, показывает, что обогащение соли йодом увеличивает ожидаемую продолжительность жизни с поправкой на ее качество [22]. Однако доказательства причинно-следственной связи между легким дефицитом йода и умственной отсталостью остаются слабыми, что затрудняет в анализ затрат и выгод потенциального улучшения интеллектуальных возможностей, связанных с коррекцией легкого дефицита йода.

По иронии судьбы, в то время, как многие беременные женщины по-прежнему остаются подвержены риску дефицита йода, в Европейских странах существует признание того, что сельскохозяйственные животные, включая молочных коров, получают несомненную пользу от адекватного потребления йода. Фактически обогащение кормов для животных йодом является устоявшейся практикой в Европейском регионе, обеспечивая адекватное потребление йода во всех секторах животноводческой отрасли и существенный экономический эффект.

Подводя итог, йодирование соли в ХХ веке предотвратило эндемический зоб и когнитивные нарушения с явными экономическими выгодами в тех странах, где дефицит йода был достаточно тяжелым. И в настоящее время потребление йода все еще не является оптимальным в значительной части Европейского региона ВОЗ, а легкий дефицит йода является причиной предотвратимой высокой частоты узлов ЩЖ и гипертиреоза. Соотношение затрат и выгод для профилактики легкого дефицита йода в Европейском регионе является благоприятным, учитывая низкую стоимость программы обогащения йодом (в Германии она оценивается примерно в 11 (евро) центов на человека в год) и значительные расходы на диагностику и лечение узлового зоба и гипертиреоза [22]. Помимо предотвращения экономических последствий легкого дефицита йода, также нельзя допустить возврата к ситуации, существовавшей в 1980-х годах, когда эндемический зоб и субклинический гипотиреоз у новорожденных были частыми явлениями.

## ЗАКЛЮЧЕНИЕ

Исследования йодного статуса в странах Европейском регионе ВОЗ указывают на в целом адекватное потребление йода школьниками, в основном за счет сочетания использования йодированной соли и йода, поступающего с молоком и молочными продуктами. Количество стран с дефицитом йода сократилось с 23 в 2003 г. до 2 в 2023 г. Обязательное йодирование соли обеспечивает адекватное потребление йода во всех группах населения, за исключением нескольких стран, где эти программы плохо реализуются. Недавние исследования указывают на снижение потребления йода в некоторых странах с добровольным йодированием соли, а понятие «легкого» дефицита йода на основе умеренно сниженной мКЙМ может быть обманчивым, поскольку предполагает наличие в популяции определенной доли населения с более тяжелым йодным дефицитом. Рутинный надзор за йодным статусом с использованием национальных репрезентативных исследований мКЙМ отсутствует в большинстве стран Европейского региона, и во многих странах имеются устаревшие данные (более 10 лет) о йодной обеспеченности. Мониторинг йодного статуса должен быть направлен на взрослое население (особенно женщин репродуктивного возраста) и/или беременных женщин. мКЙМ у детей школьного возраста может больше не отражать йодный статус для населения в целом в Европейском регионе, поскольку потребление молока и молочных продуктов (важных источников йода в странах западной и центральной Европы) у детей выше, чем у взрослых. Кроме того, во многих странах йодированная соль является обязательной для приготовления пищи в школьных столовых, но не в домохозяйствах и пищевой промышленности. Концентрация йода, измеренная в образцах разовой или суточной мочи, отражает недавнее потребление йода из всех пищевых источников и является наиболее подходящим индикатором для оценки йодного статуса населения. Существующий порог мКЙМ, определяющий адекватное потребление йода (≥100 мкг/л), был первоначально определен для детей, а затем распространен на взрослых. Однако нынешние пороговые значения мКЙМ, рекомендуемые для взрослых (более 100 мкг/л) и беременных женщин (более 150 мкг/л), могут недооценивать адекватное потребление йода в 150 мкг/день для взрослых и 250 мкг/день для беременных женщин из-за более большего объема суточной мочи (обычно ≥2 л/день) у многих взрослых популяций Европейского региона. Рекомендуется, чтобы национальный статус йода контролировался, по крайней мере, в одной группе населения каждые пять лет.

Йодирование соли (и/или опосредованное обогащение молока и молочных продуктов через использование йодных добавок к кормам у коров) при тяжелом дефиците йода предотвращает эндемический зоб и когнитивные нарушения с явными экономическими выгодами. Если в Европейском регионе не будет поддерживаться адекватное потребление йода, расстройства, связанные с дефицитом йода, могут вернуться вновь и приведут к экономическим потерям. Благоприятное соотношение затрат и выгод для профилактики легкого дефицита йода в Европейском регионе является правдоподобным, учитывая низкую стоимость программ обогащения йодом.

## ДОПОЛНИТЕЛЬНАЯ ИНФОРМАЦИЯ

Источники финансирования. Данная публикация была подготовлена при финансовой поддержке Kiwanis International и Глобальной сети по йоду.

Этот перевод не был создан Всемирной организацией здравоохранения (ВОЗ). ВОЗ не несет ответственности за содержание или точность этого перевода. Оригинальное английское издание является обязательным и аутентичным изданием: Prevention and control of iodine deficiency in the WHO European Region: adapting to changes in diet and lifestyle. Copenhagen: WHO Regional Office for Europe; 2024”.

Основными авторами доклада ВОЗ/ГСЙ являются Мария Андерссон (Maria Andersson), ГСЙ; Сара Бат (Sarah Bath), Университет Суррея; Клэр Фарранд (Clare Farrand), Европейское региональное бюро ВОЗ; Григорий Герасимов (Grigory Gerasimov), ГСЙ; Родриго Морено-Рейес (Rodrigo Moreno-Reyes), ГСЙ. Перевод на русский язык и адаптация текста выполнены профессором Г.А. Герасимовым.

Благодарности. Европейское региональное бюро ВОЗ выражает благодарность Джойс Грин (IGN), Элизабет Пирс, Лизе Роджерс (штаб-квартира ВОЗ), Вернеру Шультинку (IGN) и Майклу Циммерманну за рецензирование и предоставление ценных комментариев, а также Саре Маршанд за помощь в сборе данных, а также выражает Нармин Гулузаде (Европейское региональное бюро ВОЗ), Даниэле-Марии Мадан (Европейское региональное бюро ВОЗ), Регине Малых (Европейское региональное бюро ВОЗ) и Алене Токаревой (Европейское региональное бюро ВОЗ) за рецензирование окончательного текста доклада.

Мы признаем ценный вклад, внесенный национальными координаторами IGN, коллегами из нескольких страновых офисов ВОЗ, национальными партнерами в государствах — членах Европейского региона ВОЗ в предоставлении данных и работу по проверке информации, представленной на уровне страны/территории. Особая благодарность — Кремлину Викрамасингхе и Годену Галеа (Европейское региональное бюро ВОЗ) за общее руководство и содействие консультациям по обзору данных.

1. Этот перевод не был создан Всемирной организацией здравоохранения (ВОЗ). ВОЗ не несет ответственности за содержание или точность этого перевода. Оригинальное английское издание является обязательным и аутентичным изданием: Prevention and control of iodine deficiency in the WHO European Region: adapting to changes in diet and lifestyle. Copenhagen: WHO Regional Office for Europe; 2024”.2. Все ссылки на Косово в настоящем документе следует понимать в контексте резолюции 1244 (1999) Совета Безопасности ООН.
